# SUMOylation stabilizes hSSB1 and enhances the recruitment of NBS1 to DNA damage sites

**DOI:** 10.1038/s41392-020-0172-4

**Published:** 2020-06-24

**Authors:** Liwen Zhou, Lisi Zheng, Kaishun Hu, Xin Wang, Ruhua Zhang, Yezi Zou, Li Zhong, Shang Wang, Yuanzhong Wu, Tiebang Kang

**Affiliations:** 1 Sun Yat-sen University Cancer Center, State Key Laboratory of Oncology in South China, Collaborative Innovation Center for Cancer Medicine, Guangzhou, 510060 China; 2 Guangdong Provincial Key Laboratory of Malignant Tumor Epigenetics and Gene Regulation, Medical Research Center, Sun Yat-sen Memorial Hospital, Sun Yat‐sen University, Guangzhou, China

**Keywords:** Oncogenes, Cancer

## Abstract

Human single-stranded DNA-binding protein 1 (hSSB1) is required for the efficient recruitment of the MRN complex to DNA double-strand breaks and is essential for the maintenance of genome integrity. However, the mechanism by which hSSB1 recruits NBS1 remains elusive. Here, we determined that hSSB1 undergoes SUMOylation at both K79 and K94 under normal conditions and that this modification is dramatically enhanced in response to DNA damage. SUMOylation of hSSB1, which is specifically fine-tuned by PIAS2α, and SENP2, not only stabilizes the protein but also enhances the recruitment of NBS1 to DNA damage sites. Cells with defective hSSB1 SUMOylation are sensitive to ionizing radiation, and global inhibition of SUMOylation by either knocking out UBC9 or adding SUMOylation inhibitors significantly enhances the sensitivity of cancer cells to etoposide. Our findings reveal that SUMOylation, as a novel posttranslational modification of hSSB1, is critical for the functions of this protein, indicating that the use of SUMOylation inhibitors (e.g., 2-D08 and ML-792) may be a new strategy that would benefit cancer patients being treated with chemo- or radiotherapy.

## Introduction

The integrity of the genome is frequently challenged by various types of DNA damage induced by replication errors and/or environmental hazards. Among the different kinds of DNA damage, DNA double-strand breaks (DSBs) are the most dangerous to cells.^1–3^ Failure to repair even just one DSB can result in chromosomal fragmentation during mitosis and may induce cell death.^4^ It is therefore critical for cells to detect breaks, transduce the signal, and repair the breaks. Defects in the response to DNA damage contribute to genomic instability and tumorigenesis.^5^ There are two major mechanisms deployed by cells to repair DSBs: nonhomologous end joining (NHEJ) and homologous recombination (HR).^6^ NHEJ is a less complex form of repair than HR; in NHEJ, the two broken ends are simply joined together through ligation; therefore, this process is error prone and can be evoked during the entire cell cycle.^7–9^ However, HR depends on undamaged homologous DNA, which it uses as a template to repair DSBs; therefore, it is an error-free repair pathway and is undertaken specifically in the S or G2 phase of the cell cycle.^10^ One of the early events in HR is the activation of the ATM DNA repair kinase. This kinase initiates a signaling cascade that recruits and phosphorylates downstream repair proteins.^11^ However, ATM activation is dependent on the previous recruitment of the MRN (MRE11, RAD50, and NBS1) complex to the DSB,^12,13^ and the MRN-dependent processing of DSBs promotes the recruitment of MDC1 and the subsequent activation and maintenance of ATM kinase activity.^14–17^

Human single-stranded DNA-binding protein 1 and 2 (hSSB1 and hSSB2) are two recently identified single-stranded DNA-binding proteins in the human genome.^18^ The two proteins are structurally similar, both including a single N-terminal oligonucleotide/oligosaccharide-binding (OB)-fold domain and a conserved C-terminal domain. Interestingly, several studies have revealed that hSSB1 also plays important roles during HR, DNA replication fork repair, oxidative stress response, and cell cycle regulation.^19–23^ When DSBs occur, hSSB1 quickly translocates to the DNA damage sites, where it recruits the MRN complex and subsequent ATM through direct binding with NBS1.^24,25^ Then, the recruited ATM phosphorylates hSSB1 at threonine 117 to further augment hSSB1 stability and activity.^18^ Although the hSSB1–MRN–ATM positive feedback axis has been shown to be critical for the efficient activation of homologous repair, the precise mechanisms remain unknown.

Over the past two decades, the modification of small ubiquitin-like modifier (SUMO), a member of the ubiquitin-like protein family, has emerged as a critical factor in multiple physiological processes, including signal transduction, protein localization, and degradation, and DNA transcription, replication, and repair.^26–30^ Mammalian cells have four SUMO paralogs: SUMO1, SUMO2, SUMO3, and SUMO4. Studies have focused on SUMO1/2/3, while SUMO4 is likely not conjugated to substrates under normal physiological conditions, and its function is still unclear.^31^ Similar to ubiquitin modification, SUMO modification is also regulated through enzyme-controlled SUMOylation and deSUMOylation in a highly dynamic process. In mammals, SUMOs are activated by the SUMO-activating enzyme (E1), a heterodimer containing SAE1 and SAE2; then, SUMOs are transferred by UBC9, a SUMO-conjugating enzyme (E2); and finally, SUMOs are ligated to the ε-amino group lysine residues in substrates by SUMO E3 ligases. On the other hand, SUMOylation can be quickly reversed by SUMO/Sentrin-specific proteases (SENPs).^32^ SUMO E3 ligases confer substrate specificity, and a number of SUMO E3 ligases have been described, among which the protein inhibitor of activated STAT (PIAS) family proteins are most well studied. The PIAS family includes PIAS1, PIAS2, PIAS3 and PIAS4. There are six members of the SENP family, namely, SENP1, SENP2, SENP3, SENP5, SENP6, and SENP7.^32^

To date, many DNA damage response (DDR) proteins have been shown to be modulated by SUMOylation. In response to DNA damage, poly-SUMOylation of PARP1 induces its ubiquitination mediated by RNF4, which decreases its stability.^33^ In response to UV irradiation, the SUMOylation of TIP60 promotes its translocation from the nucleoplasm to the promyelocytic leukemia body, augments its acetyltransferase activity and promotes the p53-dependent DDR.^34^ In addition, p53 has also been shown to be SUMOylated to enhance its stability and activity.^35^ It has been shown that RAD51 interacts with UBC9 and SUMO and that defects in UBC9 result in decreased RAD51 DNA damage foci.^36^ Recently, ATRIP was also shown to be modified by SUMO2/3 at K234 and K289. SUMOylation-deficient mutants fail to localize to DNA damage sites and have compromised interactions with a number of proteins, including ATR, RPA70, and TopBP1, and the MRN complex. However, fusion of a SUMO2 chain to the ATRIP SUMOylation-deficient mutant rescues interactions of ATRIP mutant with downstream effectors, indicating that SUMOylation may provide a unique type of protein glue that enhances multiple protein interactions upon DNA damage.^37^ In addition to the aforementioned proteins, RPA70, a member of the ssDNA binding heterotrimer, has also been shown to be SUMOylated. The RPA70 SUMOylation chain may facilitate the recruitment of RAD51 molecules to DNA damage foci to initiate HR repair.^38,39^ These studies demonstrated the critical functions of the SUMOylation modification upon DNA damage through the regulation of substrate stability, activity, localization, and so on. In this study, we provide evidence that the SUMOylation of hSSB1 not only enhances its protein stability but also augments its ability to recruit NBS1 in response to DNA damage.

## Results

### hSSB1 is SUMOylated by SUMO3, and the SUMOylation of hSSB1 is enhanced in response to DNA damage

**Fig. 1:**
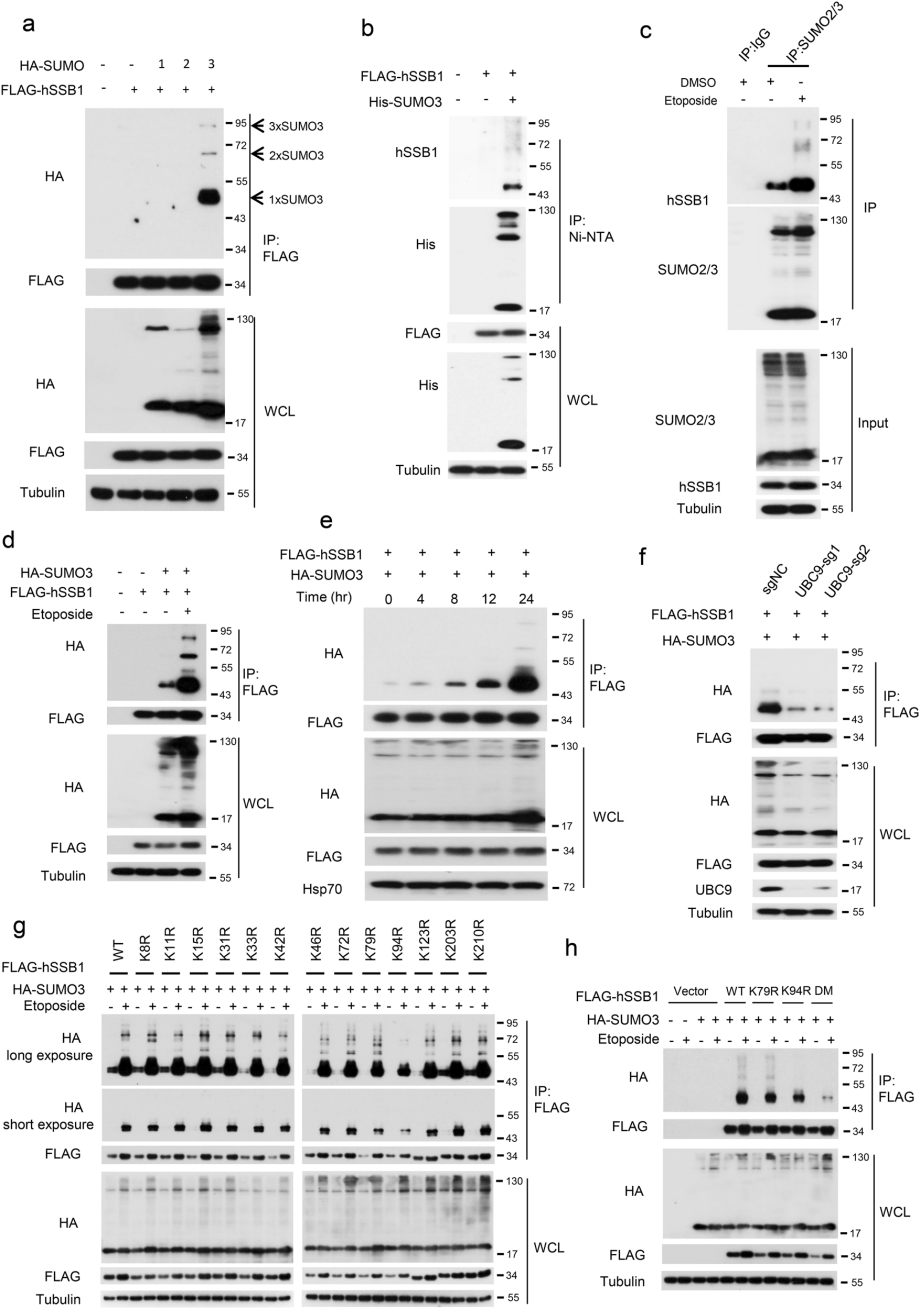
hSSB1 is SUMOylated by SUMO3. **a** Forty-eight hours after HEK293T cells were cotransfected with FLAG-hSSB1 and HA-SUMO1-3, the cells were lysed and the proteins analyzed by western blotting or immunoprecipitation (IP) using an anti-FLAG antibody followed by western blotting. WCL whole cell lysate. **b** Forty-eight hours after HEK293T cells were cotransfected with FLAG-hSSB1 and His-SUMO3, the cells were lysed. After sonication, the lysates were incubated with nickel-nitrilotriacetic acid (Ni-NTA) beads, and the pulled down proteins were analyzed by western blotting. **c** HEK293T cells treated with or without etoposide were lysed in RIPA-SDS lysis buffer, immunoprecipitated with SUMO2/3 affinity beads and the proteins, analyzed by western blotting. **d** Twenty-four hours after HEK293T cells were cotransfected with FLAG-hSSB1 and HA-SUMO3, the cells were treated with or without 100 μM etoposide, as indicated, for 24 h, and then the cells were lysed and the proteins analyzed as in **a**. **e** HEK293T cells cotransfected with FLAG-hSSB1 and HA-SUMO3 were treated with etoposide for the indicated times before harvesting, 48 h post transfection, and then the cells were lysed and the proteins analyzed as in **a**. **f** HEK293T cells with stably knocked out UBC9 were cotransfected with FLAG-hSSB1 and HA-SUMO3 for 24 h, and then treated with 100 μM etoposide for 24 h, as shown in **a**. **g**, **h** HEK293T cells were transfected with the indicated FLAG-hSSB1 plasmids with HA-SUMO3 and, after 24 h, were treated with 100 μM etoposide, as indicated, for 24 h. Then, the cells were lysed and the proteins analyzed by western blotting or IP using the anti-FLAG antibody followed by western blotting

A series of studies have revealed the critical role of hSSB1 in DNA damage; however, its regulation in response to DNA damage remains poorly understood.^18,20,40^ Since SUMOylation plays crucial roles in the response to DNA damage, we sought to determine whether hSSB1 undergoes SUMOylation. First, as shown in Fig. 1a, b, SUMOylation of ectopic hSSB1 was detected in the cells cotransfected with SUMO3 but not in the cells cotransfected with SUMO1 or SUMO2. This SUMO3-mediated SUMOylation of hSSB1 was dramatically increased at both endogenous and exogenous levels in the cells treated with etoposide, a DNA damage inducer, (Fig. 1c, d). Furthermore, the increase in the SUMOylation level caused by etoposide treatment was time dependent (Fig. 1e). Notably, mono-SUMO3 SUMOylation was dominant for hSSB1 under normal conditions and in response to DNA damage (Fig. 1a–e). Although hSSB1 and hSSB2 are highly homologous, SUMO3-mediated SUMOylation seemed to be specific for hSSB1 but not for hSSB2 (Supplementary Fig. 1). Moreover, a dramatic decrease in hSSB1 SUMOylation was observed in the cells when UBC9 was knocked out using sgRNA #1 or #2, as UBC9 is the SUMO-conjugating E2 enzyme (Fig. 1f). hSSB1 SUMOylation was impaired by SUMOylation inhibitors 2-D08 and ML-792 in a dose-dependent manner (Supplementary Fig. 2a, b).

Then, we sought to identify the SUMOylation site(s) on hSSB1. We separately mutated each individual lysine (K) in hSSB1 to arginine (R) and cotransfected these mutants or the wild type (WT) with HA-SUMO3 into HEK293T cells followed by etoposide treatment or no treatment. As shown in Fig. 1g, the mono- and poly-SUMOylation levels of K94R were dramatically decreased in response to DNA damage. In addition, a decrease in the mono-SUMOylation level of K79R was also observed in response to DNA damage. Indeed, the SUMOylation of hSSB1 was almost abolished in the K79R/K94R double-mutant (DM) cells in response to DNA damage (Fig. 1h). These results indicate that hSSB1 SUMOylation occurs at both K79 and K94, with K94 being the dominant site. Collectively, our results demonstrate that hSSB1 is SUMOylated by SUMO3 at both K79 and K94 and that this SUMOylation of hSSB1 is increased in response to DNA damage. Given that K94 of hSSB1 is the dominant site for acetylation by p300,^20^ we were curious about the relationship between SUMOylation and acetylation at this site. As shown in Supplementary Fig. 3a, b, treatment with the SUMOylation inhibitors ML-792 and 2-D08 slightly decreased the acetylation level, while treatment with the p300 inhibitor C646 simultaneously reduced the acetylation and SUMOylation levels. These results indicated that the SUMOylation and acetylation of K94 may have a synergetic effect.

### PIAS2α and SENP2 are the SUMO E3 ligase and deSUMOylation enzymes for hSSB1, respectively

**Fig. 2:**
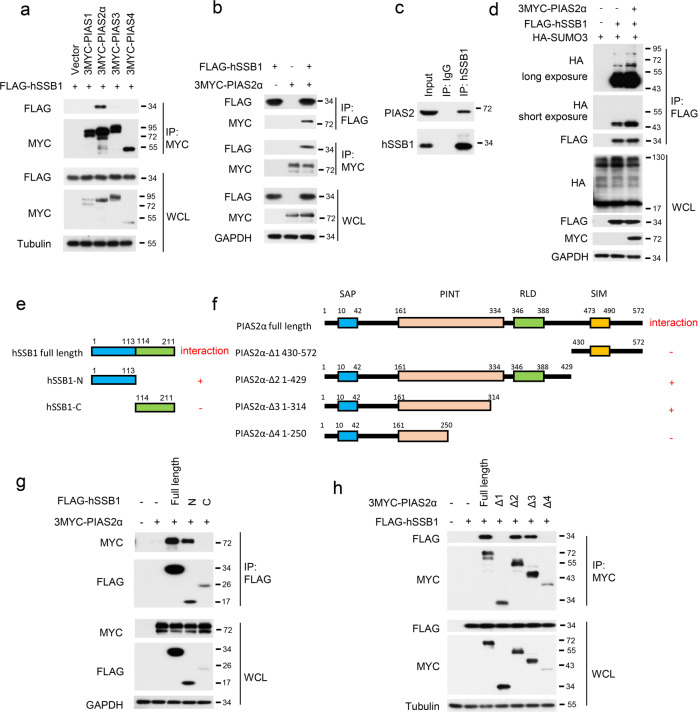
PIAS2α is the SUMO E3 ligase for hSSB1. **a**, **b** Forty-eight hours after HEK293T cells were transfected with the indicated PIAS plasmids with FLAG-hSSB1, the cells were lysed and the proteins analyzed by western blotting or IP using an anti-FLAG or anti-MYC antibody followed by western blotting, as indicated. **c** HEK293T cells were lysed with RIPA, and the lysates were subjected to IP using IgG or anti-hSSB1 antibody, as indicated, and were analyzed by western blotting. **d** HEK293T cells were cotransfected with the indicated plasmids and, after 24 h, were treated with 100 μM etoposide for 24 h, and then lysed and analyzed as in **b**. Schematic description of the hSSB1 domains (**e**) and the PIAS2α truncation constructs (**f**). **g** Cotransfection of 3MYC-PIAS2α with the indicated hSSB1 plasmids into HEK293T cells for 48 h, followed by cell lysis and analysis as in **b**. **h** Cotransfection of FLAG-hSSB1 with the indicated 3MYC-PIAS2α truncations into HEK293T cells for 48 h, followed by cell lysis and were analyzed as in **a**

Next, we sought to determine which SUMO E3 ligase is critical for the SUMOylation of hSSB1. Since the PIAS family is the most well-known SUMO E3 ligase family,^32^ PIAS1, PIAS2α, PIAS3, and PIAS4 were cloned and cotransfected individually with hSSB1 into cells. As shown in Fig. 2a, PIAS2α, but not PIAS1, PIAS3, or PIAS4, specifically interacted with hSSB1, as determined by the coimmunoprecipitation (co-IP) assay. This interaction was validated by reciprocal IP using ectopic PIAS2α and hSSB1 (Fig. 2b). Furthermore, the interaction between hSSB1 and PIAS2, both at endogenous levels, was also detected by the co-IP assay (Fig. 2c). Consistent with this finding, the hSSB1 SUMOylation level was substantially increased by the overexpression of PIAS2α in cells. These results indicate that PIAS2α is the SUMO E3 ligase for hSSB1 (Fig. 2d). Then, we mapped the domains responsible for the interaction between PIAS2α and hSSB1. As shown in Fig. 2e, f, hSSB1 was divided into two parts, an N-terminal OB-fold domain and a C-terminal domain, as previously described,^23^ and a series of PIAS2α truncation constructs were generated based on its sequence and structure. Using a co-IP assay, we found that the N-terminal OB-fold domain in hSSB1 and amino acids 250–314 in PIAS2α were the domains critical for the interaction between PIAS2α and hSSB1 in cells (Fig. 2g, h).

**Fig. 3:**
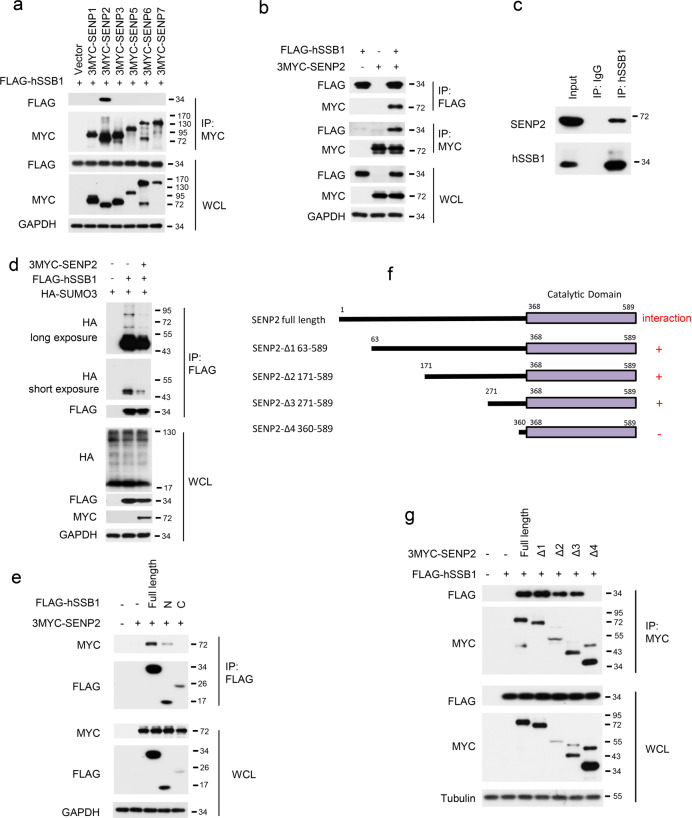
SENP2 is the deSUMOylation enzyme for hSSB1. **a**, **b** HEK293T cells were transfected with the indicated SENP plasmids with FLAG-hSSB1, lysed 48 h later and the proteins analyzed by western blotting or IP using the anti-FLAG or anti-MYC antibody followed by western blotting, as indicated. **c** HEK293T cells were lysed with RIPA, and the lysates were subjected to immunoprecipitation using IgG or anti-hSSB1, as indicated, and were analyzed by western blotting. **d** Twenty-four hours after HEK293T cells were cotransfected with the indicated plasmids, the cells were treated with 100 μM etoposide for 24 h, and then lysed and the proteins analyzed as in **b**. **e** Forty-eight hours after cotransfection of 3MYC-SENP2 with the indicated hSSB1 plasmids, the HEK293T cells followed by cell lysis and analysis as in **b**. **f** Schematic description of the SENP2 truncations. **g** Cotransfection of FLAG-hSSB1 with the indicated 3MYC-SENP2 truncations into HEK293T cells for 48 h, followed by cell lysis and analysis as in **a**

Since SUMOylation is a reversible process, quick deSUMOylation by SENPs maintains the balance between protein SUMOylation and deSUMOylation and plays critical roles in many physiological processes^32^; therefore, we investigated which SENP catalyzes the deSUMOylation of hSSB1. Upon screening the SENP family, including SENP1–3 and 5–7, only SENP2 was found to interact with hSSB1 (Fig. 3a), a finding that was validated by reciprocal IP using ectopic SENP2 and hSSB1 (Fig. 3b). The interaction between hSSB1 and SENP2 at their endogenous levels was also observed with a co-IP assay (Fig. 3c). Consistent with this finding, as shown in Fig. 3d, the overexpression of SENP2 decreased the SUMOylation level of hSSB1 in cells. These results indicate that SENP2 is the bona fide deSUMOylation enzyme of hSSB1. Interestingly, the N-terminal OB-fold domain of hSSB1 also mediates the hSSB1 interaction with SENP2 in cells (Fig. 3e). Similarly, a series of SENP2 truncation constructs were generated based on the protein sequence (Fig. 3f) and amino acids 271–360 of SENP2 and were found to be critical for the SENP2 interaction with hSSB1 in cells (Fig. 3g).

### SUMOylation of hSSB1 increases protein stability

**Fig. 4:**
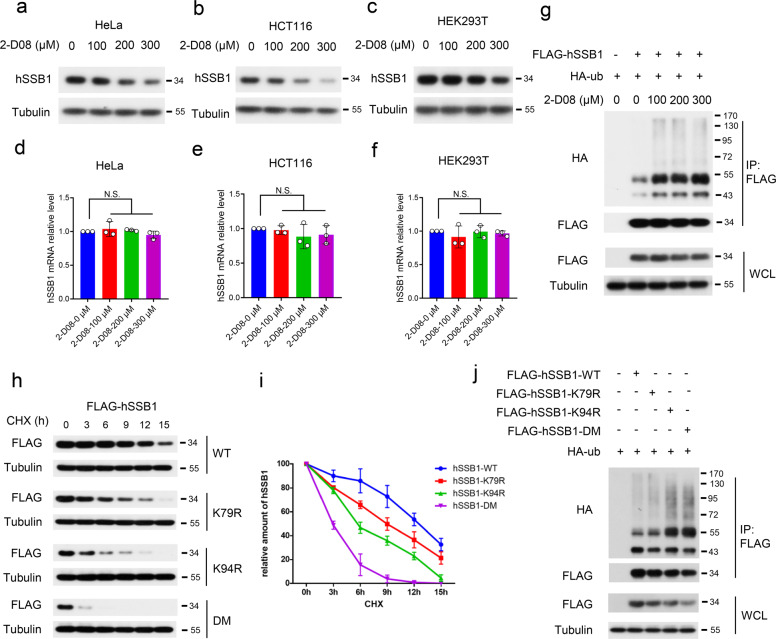
SUMOylation of hSSB1 stabilizes the protein. **a**–**f** The indicated cell lines were treated with the indicated concentrations of the SUMOylation inhibitor 2-D08, and 24 h later, the cells were subjected to western blotting or real-time PCR. **g** HEK293T cells cotransfected with FLAG-hSSB1 and HA-ub were treated 24 h later with the indicated concentrations of 2-D08 for 24 h, and then the cells were lysed and analyzed by western blotting or IP using the anti-FLAG antibody followed by western blotting. **h** HEK293T cells transfected with the indicated plasmids were treated 24 h later with 20 μg/ml cycloheximide (CHX) for the indicated times, and then the cells were lysed and analyzed by western blotting. **i** Quantification of the protein half-lives based on **h**. The results shown are averages of three independent experiments. Bars indicate the SEM. **j** HEK293T cells transfected with the indicated plasmids for 48 h were lysed and analyzed as in **g**

Given that hSSB1 is an unstable protein regulated by the ubiquitin–proteasome system^20,40^ and that SUMOylation has also been reported to participate in substrate protein stability,^32^ we surmised that SUMOylation of hSSB1 may influence its turnover. As shown in Fig. 4a–f, endogenous hSSB1 protein levels but not the corresponding mRNA levels were gradually decreased by 2-D08 in a dose-dependent manner in multiple cell lines. In accordance with this idea, the increased ubiquitination level of hSSB1 was observed in cells treated with 2-D08 (Fig. 4g). Moreover, the half-lives of WT, K79R, K94R, and K79R/K94R (DM) gradually decreased, with the DM of hSSB1 being the most unstable protein (Figs. 4h, i), while the ubiquitination levels of WT, K79R, K94R, and K79R/K94R (DM) gradually increased, with the DM of hSSB1 being the most ubiquitinated protein (Fig. 4j). In other words, the half-lives and ubiquitination levels of these hSSB1 mutants were positively and negatively correlated with their SUMOylation levels, respectively, indicating that SUMOylation of hSSB1 may somehow enhance the stability of the protein.

### SUMOylation of hSSB1 promotes its binding to NBS1 in response to DNA damage

**Fig. 5:**
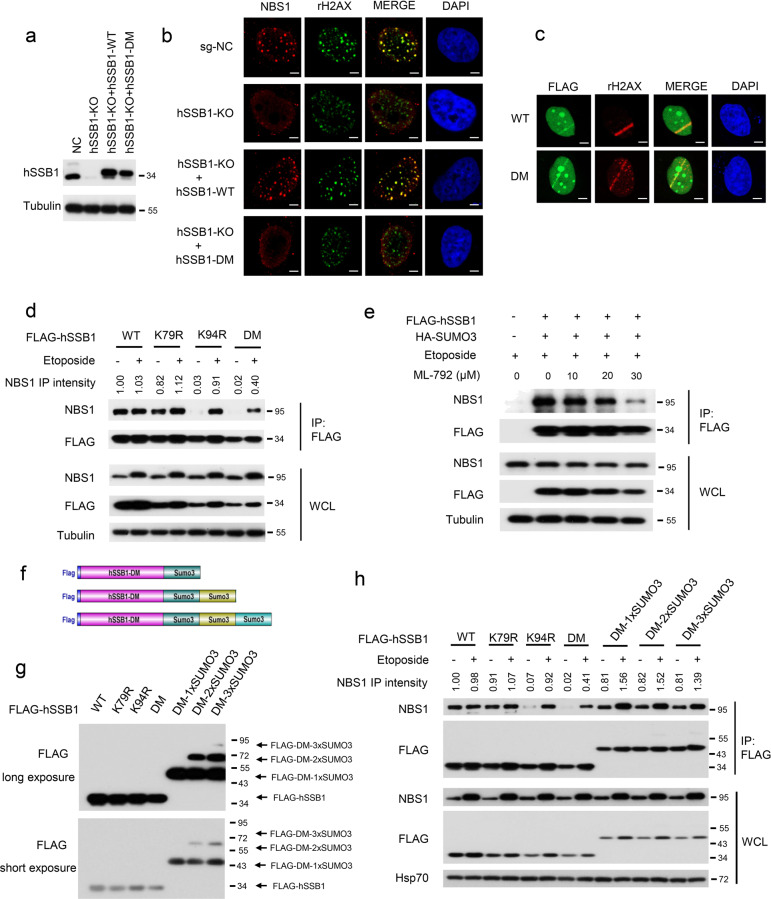
SUMOylation of hSSB1 is required for recruitment of NBS1 to DNA damage sites and enhances the binding affinity of hSSB1 to NBS1. **a** HeLa cells with stable knockout of hSSB1 by sgRNA were reintroduced with pTETON-hSSB1-WT, pTETON-hSSB1-DM, or an empty vector virus, as described in “Materials and methods,” and then the cells were lysed, and the proteins were measured by western blotting. **b** The cells in **a** were exposed to 10 Gy of gamma radiation followed by recovery for 2 h, fixed with paraformaldehyde solution and stained using the indicated antibody and DAPI; the scale bar represents 5 μm. **c** HeLa cells transfected with FLAG-hSSB1-WT or FLAG-hSSB1-DM were treated with laser microirradiation, and then stained with the indicated antibody and DAPI; the scale bar represents 5 μm. **d** HEK293T cells transfected with the indicated plasmids were treated 24 h later with or without 100 μM etoposide for 24 h, and then the cells were lysed and analyzed by western blotting or IP using the anti-FLAG antibody followed by western blotting. **e** HEK293T cells were transfected with the indicated FLAG-hSSB1 plasmid and the HA-SUMO3 plasmid for 24 h, and then treated with etoposide (100 μM) and ML-792 at the indicated concentrations for 24 h. Then, the cells were lysed and analyzed by western blotting or IP using the anti-FLAG antibody followed by western blotting. **f** Schematic illustration of FLAG-hSSB1-DM fused with 1–3 SUMO3 molecules. **g** HEK293T cells transfected with the indicated plasmids for 48 h were subjected to western blotting. **h** HEK293T cells transfected with the indicated plasmids for 24 h were treated with or without 100 μM etoposide for 24 h, and then the cells were lysed and analyzed by western blotting or IP using the anti-FLAG antibody followed by western blotting

Previous studies have shown that hSSB1 interacts with NBS1 under normal conditions and in response to DNA damage and that hSSB1 is required for the efficient recruitment of the MRN complex to DSB sites.^24,25^ Given that SUMO modification may provide a unique type of protein glue that boosts multiple protein interactions in response to DNA damage,^37^ we were very curious about whether SUMOylation of hSSB1 regulates the recruitment of NBS1 to DNA damage sites. As shown in Figs. 5a, b, knocking out hSSB1 significantly decreased NBS1 recruitment to DNA damaged foci formed in cells, which was completely rescued by reintroducing hSSB1-WT, but not hSSB1-DM, into the cells, although hSSB1-DM did not affect itself recruitment to the DNA damage foci (Fig. 5c). Furthermore, the binding of hSSB1-DM, but not hSSB1-K79R or hSSB1-K94R, to NBS1 was dramatically decreased in response to DNA damage (Fig. 5d). Consistent with this result, the SUMOylation inhibitor ML-792 also inhibited the interaction between hSSB1 and NBS1 in a dose-dependent manner (Fig. 5e). Next, we sought to rule out the possibility that the suppression of the interaction between the hSSB1 mutants and NBS1 might be due to incorrect protein folding or disruption of the hSSB1–INTS3 interaction. As shown in Supplementary Fig. 4, the mutants were unlikely to disrupt the folding of hSSB1, as shown by circular dichroism (CD) analysis and structure predictions. In addition, hSSB1-DM and hSSB1-WT treated with ML-792 did not affect the interaction between hSSB1 and INTS3 (Supplementary Fig. 5). Considering that the SUMO chain itself can interact with a series of proteins related to the DDR, including NBS1, RPA70, TopBP1, and ATR,^37^ these results indicate that SUMOylation of hSSB1 may enhance the recruitment of NBS1 to DNA damage sites in response to DNA damage.

Next, three chimeras fused to the hSSB1-DM C-terminus with one, two, or three SUMO3 chains were generated (Fig. 5f), as this fusion strategy has been used previously.^37^ Surprisingly, the mono-SUMO3 of hSSB1-DM was dominant to the chimeras fused with two or three SUMO3 chains, indicating that the poly-SUMO chains would likely be preferentially cleaved in cells at the diglycine residues between two SUMO fusion molecules (Fig. 5g). This finding was consistent with the results presented in Fig. 1, supporting the supposition that the main SUMOylation form of hSSB1 is the mono-SUMO of hSSB1 in cells. More importantly, the binding of hSSB1-DM to NBS1 was completely rescued by these chimeras under normal conditions and in response to DNA damage (Fig. 5h). Notably, the SUMO3 chimeras promoted hSSB1 interactions with NBS1, compared with the effect of hSSB1-WT, in response to DNA damage (Fig. 5h). Furthermore, since the DM-2×SUMO3 and DM-3×SUMO3 chimeras were cleaved, leaving only one SUMO3 chimera intact, we constructed a DM-3×SUMO3-no diglycine chimera, in which the diglycines were removed to protect the SUMO3 chain from being cleaved and thus retain a full-length DM-3×SUMO3 fusion protein. As shown in Supplementary Fig. 6, the DM-3×SUMO3 and DM-3×SUMO3-no diglycine chimeras showed no significant difference in their effect on hSSB1 interactions with NBS1, further validating that the mono-SUMO3 of hSSB1 is able to strengthen its interaction with NBS1. Together, these results reinforce the notion that SUMOylation of hSSB1 promotes its interaction with NBS1 in response to DNA damage.

### Targeting SUMOylation sensitizes cancer cells to agents of DNA damage

**Fig. 6:**
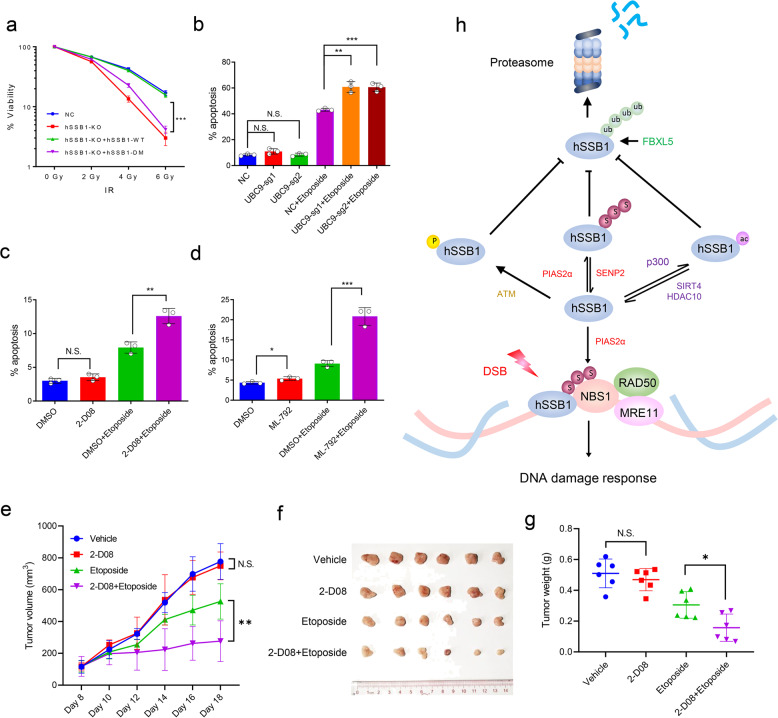
Blocking SUMOylation sensitizes cancer cells to apoptosis induced by etoposide. **a** The cells shown in Fig. 6a were treated with the indicated doses of gamma radiation, and then the cells were grown for 14 days and subjected to an analysis of cell viability, as described in “Materials and methods.” The experiments were performed in triplicate. The results shown are averages of three independent experiments. Bars indicate the SEM. ***P < 0.001, Student’s *t* test. **b** U2OS cells with UBC9 stably knocked out were seeded in six-well plates and cultured for 24 h, treated with etoposide (20 µM) for 48 h, subjected to annexin V-FITC and propidium iodide staining and measured by flow cytometry (*n* = 3). Bars indicate the SEM. **P < 0.01, Student’s *t* test. **c** HCT116 cells seeded on six-well plates and cultured for 24 h were treated with 50 µM etoposide, 200 µM 2-D08, or both for 24 h, and then were analyzed as in **b**, *n* = 3. Bars indicate the SEM. **P < 0.01, Student’s *t* test. **d** HCT116 cells seeded on six-well plates for 24 h were treated with 50 µM etoposide, 10 µM ML-792, or both for 24 h, and then were analyzed as **b**, *n* = 3. Bars indicate the SEM. **P < 0.01, ***P < 0.001 Student’s *t* test. **e**–**g** HCT116 cells were subcutaneously injected into the flanks of nude mice to generate xenograft tumors (*n* = 6/group). After 8 days of the injection, the mice were treated intraperitoneally with 2-D08 (5 mg/kg) and/or etoposide (10 mg/kg) every 2 days for 10 days. Tumor volume was measured every 2 days (**e**). After 18 days, the tumors were removed from the nude mice and weighed. Bars indicate the SEM. *P < 0.05, **P < 0.01, Student’s *t* test. **h** Proposed model for the posttranslational regulation of hSSB1. In the case of DNA damage, hSSB1 is phosphorylated by ATM, acetylated by p300 and SUMOylated by PIAS2α. These three modifications prevent its ubiquitination and degradation by FBXL5. The SUMOylation of hSSB1 promotes the recruitment of NBS1 by hSSB1 to DNA damage sites to execute its functions in response to DNA damage. Both SIRT4 and HDAC10 are critical for the deacetylation of hSSB1, while SENP2 mediates the deSUMOylation of hSSB1

Finally, we explored the biological significance of hSSB1 SUMOylation. It has been reported that knocking down hSSB1 increases chemo- and radiosensitivity.^18,20,23^ As shown in Fig. 6a, knocking out hSSB1 in cells enhanced the sensitivity of these cells to ionizing radiation, which was completely rescued by reintroducing hSSB1-WT, but not hSSB1-DM, into the cells. This result indicates that SUMOylation of hSSB1 is critical for cell survival under DNA damage conditions. In fact, as mentioned previously, many proteins, including PARP1, RPA70, TopBP1, ATR, ATRIP, BRCA1, and MDC1,^33–35,37,38^ have also been shown to be SUMOylated in response to DNA damage, and the SUMOylation of these proteins plays important roles in DNA repair and cell survival.^31^ This finding prompted us to test whether knocking out UBC9 or treating cells with 2-D08 and ML-792, which decreases global SUMOylation levels, would enhance the sensitivity of cancer cells to the chemotherapy drugs commonly used in the clinic, such as etoposide. Interestingly, we found that knocking out UBC9 with two highly efficient lenti-CRISPR sgRNAs did not affect the apoptosis rate in the short term but increased the apoptosis of cancer cells treated with etoposide (Fig. 6b). Apoptosis was also enhanced in cancer cells when treated with a combination of 2-D08 or ML-792 with etoposide compared with the treatment of etoposide alone, while 2-D08 or ML-792 treatments administered alone only slightly affected apoptosis (Fig. 6c, d). Furthermore, as shown in Fig. 6e–g, the combination of etoposide and 2-D08 sensitized tumors in nude mice to etoposide. These results indicate that targeting SUMOylation enhances the sensitivity of cancer cells to DNA damage agents.

## Discussion

In this report, we revealed that the SUMOylation of hSSB1 adds a novel layer of regulation that not only stabilizes the protein but also serves as a protein glue for recruiting NBS1 to DNA damage sites in response to DNA damage. Combined with other reports,^18,20,40^ as shown in Fig. 6h, we propose that multiple posttranslational modifications, such as phosphorylation, acetylation, ubiquitination, and SUMOylation, play key roles in the functions of hSSB1, mainly by stabilizing the protein, efficiently recruiting NBS1, and sustaining genome integrity.

hSSB1 is an evolutionarily conserved single-stranded DNA-binding protein, and its posttranslational modifications have been investigated in several laboratories, including that of our group.^18,20,40^ Initially, hSSB1 was shown to be phosphorylated by ATM at T117 to stabilize the protein and enhance its functions.^18^ The E3 ubiquitin ligase FBXL5 mediates the degradation of hSSB1 by the ubiquitin–proteasome system.^40^ In our previous work, we showed that the acetylation of the hSSB1 protein at K94 enhances its stability by inhibiting its ubiquitination and degradation.^20^ Interestingly, the K94 acetylation site is also the dominant SUMOylation site, as reported here. This finding indicates that acetylation and SUMOylation may participate in crosstalk or have synergistic effects on hSSB1 stability under normal conditions and in response to DNA damage. As shown in Fig. 4h, the mutation of K79 (the minor SUMOylation site) and K94 (the acetylation site and the major SUMOylation site) decreased the hSSB1 half-life moderately and dramatically, respectively; the K79R/K94R double mutant of hSSB1, lacking both acetylation and SUMOylation capacity, had the shortest half-life. It has been reported that, among different posttranslational modifications, SUMOylation levels are particularly low because only a small percentage of any protein undergoes this modification and because this modification is based on a highly dynamic reversible conjugation.^41,42^ Since the acetylation level of hSSB1 is relatively high under both normal and DNA damage conditions,^20^ we speculate that the acetylation of hSSB1 at K94 may contribute mainly to protein stability, while SUMOylation of hSSB1 at both K79 and K94 mainly enhances hSSB1 recruitment of NBS1 to DNA damage sites, where it execute its functions in response to DNA damage. We speculate that the mutation K79R, K94R, or both in hSSB1 likely does not affect its structure or function on the basis of the following evidence: (1) the CD spectra of hSSB1-WT and mutants are not significantly different, indicating that the mutants have the correct protein secondary structures (Supplementary Fig. 4a). (2) The predicted structures of hSSB1-WT and its DM mutant (hSSB1-DM), as shown in Supplementary Fig. 4b, were not significantly different. (3) hSSB1-DM did not affect the foci formation after IR, as shown in Fig. 5c. Huang et al. indicated that the SOSS and MRN complexes may act together, in part, through the INTS3 and NBS1 interaction,^43^ while Richard et al. demonstrated that hSSB1 interacts directly with NBS1 and stimulates MRN recruitment to DSBs.^24^ Our data showed that hSSB1-DM did not affect foci formation after IR (Fig. 5c), and both hSSB1-DM and hSSB1-WT in cells treated with ML-792 showed dramatically attenuated binding with NBS1 but not with INTS3 (Fig. 5d, e and Supplementary Fig. 5). It is worth noting that co-IP, such as in the hSSB1-DM and INTS3 assay, does not provide direct evidence for binding, while evidence from isothermal calorimetry (ITC), surface plasmon resonance (SPR), or other biophysical methods can be performed to directly and quantitatively assess the binding of hSSB1-DM and INTS3. Huang et al. have shown that INTS3 is required for hSSB1 DNA damage foci formation. Based on our observations, the hSSB1 foci were not disrupted by the mutants, indicating that the INTS3–hSSB1 interaction and function were not affected. These results support the conclusion that the inability of NBS1 recruitment was a result of the decreased hSSB1 interaction but not an indirect result of INTS3. Similar to acetylation,^20^ SUMOylation seems to be specific for hSSB1 but not its homolog, hSSB2 (Supplementary Fig. 1). These observations indicate that the regulation of hSSB1 is more controlled and sophisticated than that of hSSB2, explaining why hSSB1 plays more important roles than hSSB2 in the response to DNA damage.^44^

SUMOylation/deSUMOylation can elicit rapid and reversible biological changes through the dynamic alteration of biochemical properties of a substrate.^32^ In this report, PIAS2α and SENP2 were identified as the dominant enzymes regulating the SUMOylation and deSUMOylation of hSSB1, respectively. This is the first report to show that PIAS2α is involved in the DDR pathway via SUMOylation of hSSB1, although PIAS2α has been shown to have many substrates, such as androgen receptor, c-Jun, p53, Elk-1, PTEN, and α-synuclein.^45–49^ This finding is very important, as the SUMOylation of hSSB1 by PIAS2α enhances the recruitment of NBS1 to DNA damage sites, which is required for hSSB1 to execute its functions in response to DNA damage (Figs. 5b and 6a). On the other hand, the function of SENP2 in DNA damage has been previously reported. For instance, SENP2 promotes DNA damage signaling through its protease activity to supply or redistribute SUMO molecules, since HR repair has a great need for SUMO conjugates.^50^ Genotoxic stress activates SUMOylation of NEMO, which leads to IKK activation, and then activated NFκB promotes the expression of SENP2, which serves as the primary deSUMOylation protease for NEMO, subsequently leading to feedback attenuation of IKK and NFκB activation.^51^

The MRN complex contains a single NBS1 protein, a flexible MRE11 dimer and two RAD50 subunits, which maintain the bond between the two ends of the DNA break.^52,53^ For a long time, the MRN complex was thought to be the first repair apparatus localized to DSB sites; however, a recent study revealed that hSSB1 is required for MRN recruitment to the site.^4,24,25^ After DSBs are induced, hSSB1 binds to and sequesters the ssDNA exposed at the ends of breaks and then recruits the MRN complex to resect the duplex DNA by extending the ssDNA. The newly produced ssDNA recruits increased amounts of hSSB1 and MRN to the break sites, thus forming positive feedback for the initiation of DNA repair.^4^ In this process, the quick and efficient recruitment of NBS1 by hSSB1 is one of the key events; however, the mechanism by which hSSB1 recruits NBS1 remains unknown. In this report, we demonstrated that the SUMOylation of hSSB1 is required for the efficient recruitment of NBS1 by hSSB1 during this event (Fig. 5b–h), as it seems to kill two birds with one stone. SUMOylation of hSSB1 at both K79 and K94 stabilizes the protein (Fig. 4h); the SUMO molecules conjugated on hSSB1 provide additional docking sites with high binding affinity for NBS1 (Fig. 5d, e, h), which in turn augments the DDR cascade.

The DDR cascade is a fine-tuned signaling pathway, as reflected by the fact that DDR proteins are recruited in an orderly manner and activated at DNA damage sites. Multiple posttranslational modifications are critical for this orchestrated activation, including phosphorylation, ubiquitination, poly(ADP-ribosylation), acetylation, and methylation.^8^ In addition to the aforementioned modifications, SUMOylation has also been shown to participate in the DDR and confer genome stability.^54^ Furthermore, recent screens also revealed that SUMO substrates are highly enriched for regulators of DNA metabolism,^55,56^ highlighting the fact that SUMOylation preserves genome integrity in coordination with other posttranslational modifications. As shown in Fig. 6a, cells with defects in hSSB1 SUMOylation at both K79 and K94 are very sensitive to ionizing radiation. Since multiple other DDR proteins undergo SUMOylation, which is essential for maintaining genome integrity,^54^ we speculate that blocking SUMOylation may enhance cancer cell sensitivity to agents of DNA damage. Indeed, knocking out UBC9 or treating cancer cells with 2-D08 and ML-792 significantly enhanced the apoptosis induced by etoposide (Fig. 6b–d); more importantly, the combination of etoposide with 2-D08 sensitized tumor cells to etoposide in vivo (Fig. 6e–g). It is worth mentioning that, in addition to SUMOylation, 2-D08 inhibits Axl, IRAK4, ROS1, MLK4, GSK3β, RET, KDR, and PI3Kα in biochemical kinase assays in vitro,^57^ which may also contribute to the inhibitory effect of 2-D08 on tumors when it was combined with etoposide in vivo. Collectively, our findings argue that SUMOylation inhibitors (e.g., 2-D08 and ML-792) may be potentially combined with chemo- or radiotherapy to benefit patients with cancer.

## Materials and methods

### Cells and reagents

HeLa, HEK293T, U2OS, and HCT116 cells were maintained in Dulbecco’s modified Eagle’s medium (Life Technologies) supplemented with 10% fetal bovine serum (Life Technologies) with 5% CO_2_ at 37 °C. All cell lines used in this study were authenticated using short-tandem repeat profiling less than 6 months ago, when this project was initiated, and the cells were not cultured for more than 2 months. The cells were mycoplasma negative and tested by PCR monthly.

### Plasmid construction

FLAG-hSSB1 and the related K-to-R mutants were cloned into a pCDNA3.1 vector. FLAG-hSSB1-DM-1/2/3×SUMO3, HA-SUMO1, HA-SUMO2, HA-SUMO3, His-SUMO3, PIASs, SENPs, and related truncation constructs were cloned into a pSIN vector through infusion. For FLAG-hSSB1-DM-1/2/3×SUMO3, the SUMO3 C-terminal GG was retained, and for the FLAG-hSSB1-DM-3×SUMO3-no diglycine mutant, the diglycines were removed through infusion. For protein purification, hSSB1 and the related K-to-R mutants were cloned into a pGEX-6p vector. pTETON-hSSB1/DM was cloned for doxycycline-inducible expression of hSSB1/DM. Sequences targeting UBC9 and hSSB1 were cloned into a lenti-CRISPR V2 plasmid (Addgene, 52961): UBC9-sg1: ACATTCGGGTGAAATAATGG; UBC9-sg2: TAGAGGAGGACAAGGACTGG; and hSSB1: TGAGGTTCGGACCTGCAAAG. HA-ub was a gift from Prof. Helen Piwnica-Worms (The University of Texas MD Anderson Cancer Center).

### Western blot analysis

Cells were lysed in RIPA lysis buffer (50 mM Tris–HCl, 150 mM NaCl, 5 mM EDTA, 0.5% Nonidet P-40, and a protease and phosphatase inhibitor cocktail (Calbiochem)). Proteins were separated by SDS-PAGE and transferred into 0.45 μm PVDF membranes (Millipore). The immunoblots were processed according to standard procedures using primary antibodies against GAPDH (CST, 2118), HA (CST, 3724), FLAG (CST, 14793), MYC (CST, 2272), His (CST, 9991), UBC9 (CST, 4786), rH2AX (CST, 9718), Hsp70 (Santa Cruz, sc-24), hSSB1 (Bethyl, A301–938A), NBS1 (GeneTex, GTX70224), tubulin (Bioworld, BS1482M), PIAS2 (GeneTex, GTX115180), SENP2 (GeneTex, GTX110504), INTS3 (Bethyl, A300-427A), and SUMO2/3-HRP (ASM23-HRP, Cytoskeleton).

### Immunoprecipitation

For exogenous immunoprecipitation, HEK293T cells transfected with the indicated plasmids were lysed in RIPA lysis buffer, and then centrifuged at 12,000 rpm for 30 min. The supernatants were first incubated overnight with anti-FLAG-agarose or anti-MYC-agarose (Sigma Chemical Co.) at 4 °C, and the precipitates were washed five times with RIPA lysis buffer before immunoblotting was performed. For endogenous immunoprecipitation, the HEK293T cells were lysed in RIPA lysis buffer and centrifuged at 12,000 rpm for 30 min. The clarified supernatants were first incubated with anti-hSSB1 antibody for 2 h at 4 °C. Then, protein A/G-agarose was added and allowed to incubate overnight, and the precipitates were washed four times with RIPA and analyzed by western blotting. For nickel-nitrilotriacetic acid (Ni-NTA) bead immunoprecipitation, HEK293T cells grown to 80% confluence were transfected with His-SUMO3 and the indicated constructs. After 48 hours of transfection, the cells were lysed in buffer A (6 M guanidine-HCl, 0.1 M Na2HPO4/NaH2PO4, and 10 mM imidazole [pH 8.0]). After sonication, the lysates were incubated with Ni-NTA beads (QIAGEN) for 3 h at room temperature. Subsequently, the His pull-down products were washed twice with buffer A, twice with buffer A/TI (1 volume buffer A to 3 volumes buffer TI), and once with buffer TI (25 mM Tris–HCl and 20 mM imidazole [pH 6.8]). The pull-down proteins were resolved in 5× SDS-PAGE loading buffer and subjected to immunoblotting. For the endogenous SUMOylation assay, 2 × 10^7^ cells were lysed in 1 ml of RIPA-SDS lysis buffer (50 mM Tris–HCl, 150 mM NaCl, 0.1% SDS, 1% Triton, 0.5% sodium deoxycholate, 10 mM N-ethylmaleimide, protease inhibitors and phosphatase inhibitors [pH 8.0]). The viscous lysate was sonicated until it became fluid. SUMO2/3 affinity beads (ASM24, Cytoskeleton) were added to the lysates, and the tubes were incubated on a rotating platform at 4 °C for 4 h. The beads were collected by centrifugation at 5000 × *g* for 1 min at 4 °C, and as much supernatant as possible was aspirated without disturbing the beads. The beads were washed five times in 1 ml of lysis buffer. After the final wash, the buffer was removed completely, and 30 μl of bead elution buffer (BEB01, Cytoskeleton) was added and retained for 5 min. Then, the supernatant was collected, and 2 μl of β-mercaptoethanol was added, followed by mixing well and boiling for 5 min. The SUMOylated proteins were detected by western blotting with hSSB1 antibody and SUMO2/3-HRP antibody (ASM23-HRP, Cytoskeleton).

### RNA extraction and real-time qPCR

Total RNA was extracted from samples (RNAprep pure cell/bacteria kit; TIANGEN), quantified by using a Nanodrop 2000 spectrophotometer and stored at −80 °C. One microgram of RNA was reverse transcribed using a HiScript II 1st Strand cDNA synthesis kit (Vazyme) following the manufacturer’s recommendations. Transcripts were quantified by real-time qPCR using a LightCycler 480 instrument (Roche Diagnostics) and ChamQ SYBR qPCR Master Mix (Vazyme) according to the manufacturer’s instructions. The following qPCR primers were used:

hSSB1-F: TCTGTCTGGGACGATGTTG

hSSB1-R: GTTTGGCTCACTGAAGTTAGG

GAPDH-F: TGACTTCAACAGCGACACCC

GAPDH-R: CTGGTGGTCCAGGGGTCTTA

### Cell survival assays

HeLa cells were infected with lentivirus encoding the hSSB1 CRISPR-Cas9 sequence and then reinfected with pTETON-hSSB1/DM or an empty vector virus. The WT/DM were same sense mutated to be resistant to CRISPR-Cas9 cleavage, after. After puromycin selection for 7 days, the cells were treated with doxycycline (5 ng/ml) to induce the expression of WT/DM. Then, 500 cells were seeded into six-well plates for 24 h, and the cells were irradiated at 2, 4, or 6 Gy as indicated. The cells were then incubated for 14 days. The resulting colonies were fixed and stained with crystal violet.

### Immunofluorescence

Cells cultured on glass bottom wells were exposed to 10 Gy of gamma radiation followed by recovery for 2 h. The cells were then fixed using a 3% paraformaldehyde solution for 10 min at room temperature, and then treated with buffer containing 0.2% Triton X-100 for 10 min. The samples were blocked with 5% goat serum for 30 min and incubated with primary antibody for 2 h at room temperature. The samples were washed three times and incubated with secondary antibody for 30 min. The cells were then counterstained with DAPI to visualize nuclear DNA.

### Protein purification

pGEX-6p-hSSB1 vectors were expressed in *E. coli* Rosetta (DE3) cells. The transformed bacteria were cultured at 37 °C before induction with 0.1 mM isopropyl-1-thio-β-d-galactopyranoside at an OD 0.6 at 600 nm and grown for 16 h at 18 °C in LB medium. The cells were harvested, resuspended in PBS with protease inhibitor cocktail (Merck), and lysed using a cell disruptor (JNBIO). After centrifugation, the clarified cell lysate was incubated with GST-BIND Resin (Merck) and washed with wash buffer (PBS, 400 mM NaCl, and 1 mM DTT [pH 7.4]). The GST tag was removed by GST-PreScission protease (Beyotime Biotechnology) via on-bead cleavage, and the released hSSB1 proteins were quantified using the Bradford method.

### Circular dichroism spectroscopy

Proteins were diluted to 0.2 mg/ml in ddH_2_O and subjected to CD measurements using a Chirascan spectrometer (Applied Photophysics) in quartz cuvettes with a path length of 0.5 mm. The spectra were recorded from 190 to 260 nm at a bandwidth of 1 nm. All data were collected using a stop resolution of 1 nm and time per point of 0.5 s. A control spectrum obtained from diluted buffer was subtracted from the original data. CD readouts were converted to mean residue ellipticity.

### Laser microirradiation

DSBs were introduced in the nuclei of cultured HeLa cells by microirradiation and observed using a Zeiss Axiovert equipped with an LSM 520 Meta microscope. Briefly, cells cultured on 35-mm glass bottom dishes transfected with FLAG-hSSB1-WT or FLAG-hSSB1-DM were maintained with 10 µM BrdU for 24 h. A 365-nm laser was used to generate BrdU-dependent DSBs along the laser track. The output of the laser was set to 10 pulses and 50% transmission. After 10 min, the cells were pre-extracted after a 5-min incubation in buffer containing 0.5% Triton X-100 and fixed with 3% paraformaldehyde for 10 min at room temperature. The cells were then incubated with FLAG and rH2AX antibodies for 1 h at room temperature. Following three 5-min washes with PBS, the cells were incubated with the indicated secondary antibodies for 30 min. The cells were also counterstained with DAPI to visualize nuclear DNA.

### Apoptosis assay

U2OS cells with stably knocked out UBC9 were seeded on six-well plates for 24 h, and then treated with etoposide (20 µM) for 48 h to induce apoptosis. Then, the cells were collected by trypsin without EDTA, washed with PBS, subjected to annexin V-FITC, and propidium iodide staining according to the manufacturer’s recommendations (KeyGen Biotech), and analyzed by flow cytometry. HCT116 cells were treated with 50 µM etoposide, 200 µM 2-D08, 10 µM ML-792, or a combination of these drugs, as indicated, for 24 h, and then the cells were collected and analyzed by flow cytometry.

### Statistical analysis

All statistical experiments were performed independently and in triplicate. Statistical analysis was carried out using GraphPad Prism software (version 6.0). Data are shown as the mean ± SEM. A *p* value < 0.05 indicated a significant difference.

### Xenograft tumor model

Animal studies were approved by the Animal Research Committee of Sun Yat-sen University Cancer Center. Male athymic BALB/C nude mice (4 weeks old) were obtained from Vital River Laboratory Animal Technology (Beijing, China). Briefly, 4 × 10^6^ HCT116 cells were resuspended in 0.1 ml of PBS and subcutaneously injected into the flanks of the mice. After 8 days of the injection, the mice were treated intraperitoneally with 2-D08 (5 mg/kg) and/or etoposide (10 mg/kg) every 2 days for 10 days. The solvents used were 5% DMSO, 40% PEG300, and 55% normal saline. Tumor volumes were measured every 2 days and were calculated using the formula *V* = 0.5 × length × width^2^. All mice were sacrificed 18 days after injection, and the xenograft tumors were isolated, photographed, and weighed.

## Supplementary information

Supplementary Materials

## Data Availability

The data sets used for the current study are available from the corresponding author upon reasonable request.
