# Supplementary material for: SUMOylation stabilizes hSSB1 and enhances the recruitment of NBS1 to DNA damage sites
Source: Signal Transduct Target Ther. 2020 Jun 24;5:80. doi: 10.1038/s41392-020-0172-4 (PMC7311467; doi:10.1038/s41392-020-0172-4)
Supplement: Supplementary file 1 — Supplementary Materials [file 41392_2020_172_MOESM1_ESM.pdf]

Supplementary Materials for

SUMOylation stabilizes hSSB1 and enhances the recruitment of NBS1 to DNA damage sites

Liwen Zhou[1 *], Lisi Zheng[1 *], Kaishun Hu[2 *], Xin Wang[1], Ruhua Zhang[1], Yezi Zou[1], Li Zhong[1], Shang Wang[1], Yuanzhong Wu[1 #] and Tiebang Kang[1 #]

Correspondence to: kangtb@sysucc.org.cn; wuyzh@sysucc.org.cn

This PDF file includes:

Figures. S1 to S6

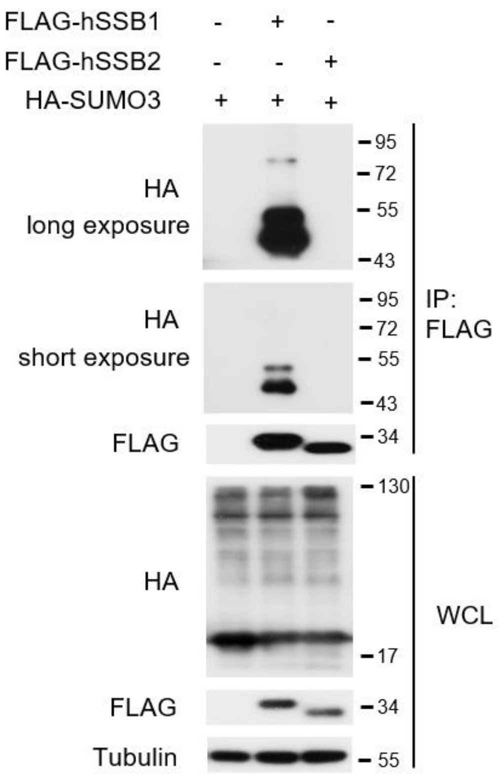

Figure. S1.

SUMOylation of hSSB1, but not of hSSB2, was detected.

HEK293T cells were cotransfected with FLAG-hSSB1 or FLAG-hSSB2 and HA-SUMO3 for 24 hr and then treated with 100 μM etoposide for 24 hr. Then, the cells were lysed and analyzed by Western blotting or immunoprecipitation (IP) using an anti-FLAG antibody followed by Western blotting. WCL: whole cell lysate.

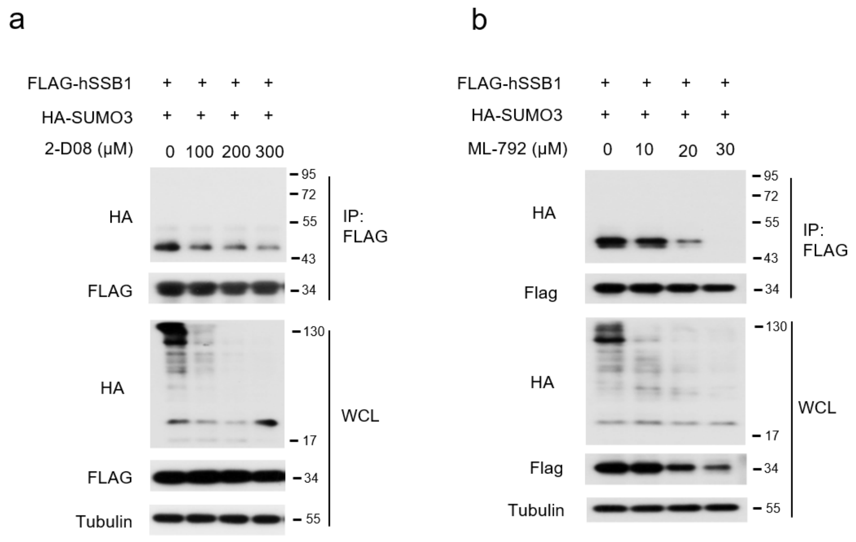

Figure. S2.

hSSB1 SUMOylation was impaired by the SUMOylation inhibitors 2-D08 and ML-792. HEK293T cells were cotransfected with FLAG-hSSB1 and HA-SUMO3 for 24 hr and then treated with the indicated concentrations of 2-D08 **(a)** or ML-792 **(b)** for 24 hr. Then, the cells were lysed and analyzed by Western blotting or immunoprecipitation (IP) using the anti-FLAG antibody followed by Western blotting. WCL: whole cell lysate.

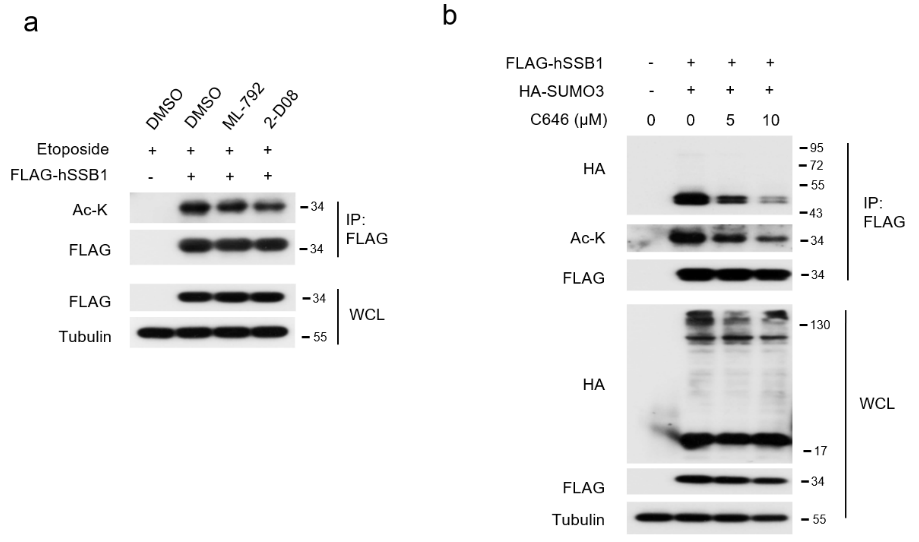

Figure. S3.

Relationship between SUMOylation and acetylation of hSSB1.

(a) HEK293T cells were transfected with the indicated FLAG-hSSB1 plasmid for 24 hr and then treated with etoposide (100 μM) as well as DMSO, ML-792 (10 μM), or 2-D08 (100 μM) as indicated for 24 hr. Then, the cells were lysed and analyzed by Western blotting or IP using the anti-FLAG antibody followed by Western blotting. **(b)** HEK293T cells were transfected with the indicated FLAG-hSSB1 plasmid and the HA-SUMO3 plasmid for 24 hr and then treated with etoposide (100 μM) and C646 as indicated for 24 hr. Then, the cells were lysed and analyzed by Western blotting or IP using the anti-FLAG antibody followed by Western blotting.

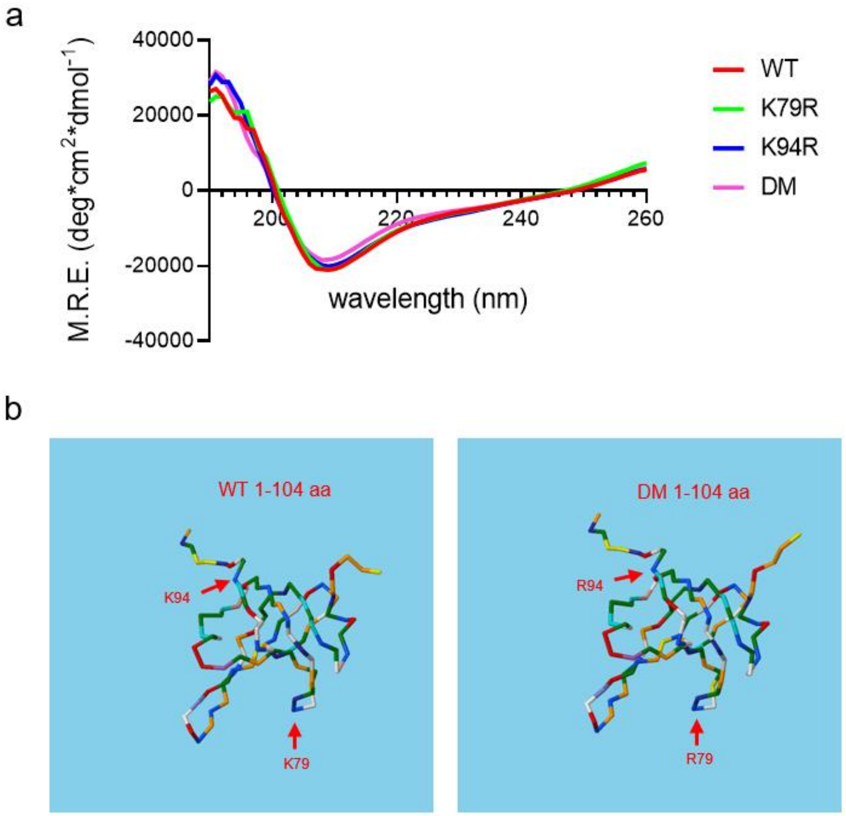

Figure. S4.

Circular dichroism spectra and predicted structures of hSSB1-WT and related mutants.

(a) CD spectra of hSSB1 WT and related mutants. M.R.E. denotes the mean residue ellipticity.

(b) 1-104 aa of hSSB1-WT and hSSB1-DM structures were predicted by RaptorX. WT P-value: 4.75e-07, DM P-value: 4.19e-07.

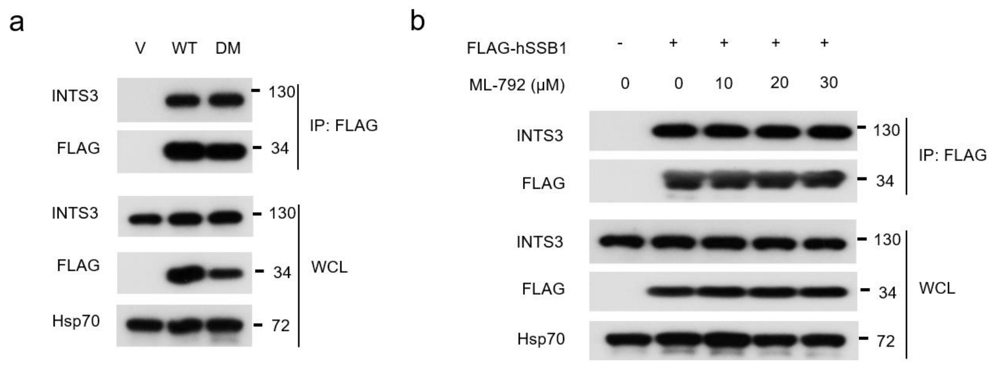

Figure. S5.

hSSB1-DM and the SUMOylation inhibitor ML-792 treatment did not affect the interaction between hSSB1 and INTS3.

(a) HEK293T cells were transfected with the indicated plasmids for 24 hr and then treated with etoposide (100 μM) for 12 hr. Then, the cells were lysed and analyzed by Western blotting or IP using the anti-FLAG antibody followed by Western blotting. **(b)** HEK293T cells were transfected with the FLAG-hSSB1 plasmid for 24 hr and then treated with etoposide (100 μM) and the indicated concentrations of ML-792 for 12 hr. Then, the cells were lysed and analyzed by Western blotting or IP using the anti-FLAG antibody followed by Western blotting.

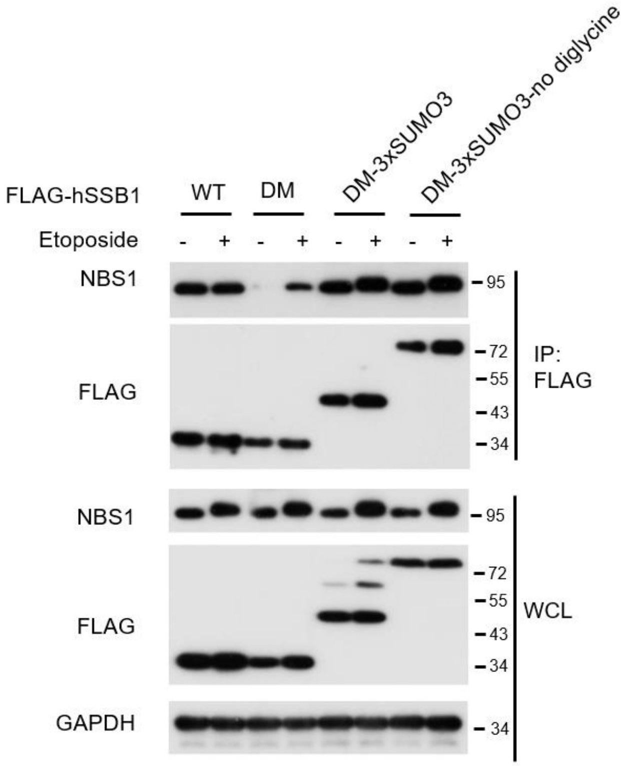

Figure. S6.

DM-3xSUMO3 and DM-3xSUMO3-no diglycine chimeras showed no significant difference in their interactions with NBS1.

HEK293T cells were transfected with the indicated plasmids for 24 hr and then treated with etoposide (100 μM) for 12 hr. Then, the cells were lysed and analyzed by Western blotting or IP using the anti-FLAG antibody followed by Western blotting.
